# Consumer Perceptions and Purchase Behavior Towards Plant-Based Eggs: A Vignette Experiment

**DOI:** 10.3390/foods14101742

**Published:** 2025-05-14

**Authors:** Da Eun Kim, Brenna Ellison

**Affiliations:** 1 Department of Agricultural and Consumer Economics, University of Illinois Urbana-Champaign, Urbana, IL 61801, USA; daeunek2@illinois.edu; 2 Department of Agricultural Economics, Purdue University, West Lafayette, IN 47907, USA

**Keywords:** plant-based eggs, purchase likelihood, consumer perception, vignette

## Abstract

The plant-based eggs category is growing rapidly; however, there is limited research on consumer behavior towards plant-based eggs. We explore consumer perceptions and investigate factors affecting the likelihood of purchase for plant-based eggs by using experimental vignettes. Using data from a nationally representative sample of U.S. consumers, we find that traditional eggs were rated more favorably than plant-based eggs, though plant-based eggs were perceived more favorably on environmental impact and animal welfare for food-related attributes and on total fat and cholesterol for nutrition components. Additionally, consumers were more likely to purchase plant-based eggs at lower prices and when served as an ingredient to a main dish (pancakes) rather than on their own (scrambled eggs).

## 1. Introduction

Current food systems, which encompass interlinked activities from food production to consumption, have a significant impact on the environment. It is estimated that global food systems are responsible for more than one-third of global anthropogenic greenhouse gas emissions [1]. Among various activities within food systems, livestock production and consumption is identified as one of the leading factors contributing to negative environmental impacts [2]. In the context of the poultry industry, Abiín et al. [3] found that intensive egg production has a substantial negative impact on natural land transformation, terrestrial ecotoxicity, and freshwater ecotoxicity, with a carbon footprint of 2.66 kg CO_2_ equivalent per dozen eggs produced. Further, there has been a push to reduce consumption of animal-based foods as a potential means to address environmental and animal welfare concerns, as well as to alleviate public health concerns [4]. The growing awareness and interest in these issues has resulted in the introduction of many new plant-based foods that are argued to be more sustainable (and often, equivalent or better nutritionally) than their conventional, animal-based counterparts.

According to the industry reports, such as the Plant Based Foods Association [5] and the Good Food Institute [6], 2023 U.S. retail sales of plant-based food reached USD 8.1 billion, a 6.6% increase from 2021. This sales growth happened across a variety of plant-based food categories, including eggs. Plant-based eggs are alternatives to traditional eggs made entirely from plant-based ingredients such as pulses and legumes, cereals, or algae. Sales of plant-based eggs have shown consistent growth over the past years. Between 2019 and 2020, plant-based eggs sales increased by 173%, followed by a 43% increase from 2020 to 2021 and a 14% increase from 2021 to 2022, reaching USD 45 million [5]. This upward trend continued, with sales reaching USD 53.1 million in 2023 and USD 71.1 million in 2024, and the market is projected to grow substantially in the future [7]. While the plant-based eggs category constitutes a relatively minor share (0.5%) of the total U.S. egg market, it is considered one of the fastest growing segments in the plant-based food categories [8]. More recently, plant-based eggs sales have benefitted from egg shortages caused by avian flu outbreaks, which has made traditional eggs less available and more expensive [9]. Combined with consumers’ growing interest in health and wellness, these market conditions have contributed to the rapid expansion of plant-based eggs sales.

Despite the growing sales in the plant-based eggs market, there is limited research regarding consumer behavior towards plant-based eggs. Much of the current literature focuses on consumer perceptions of and preferences/demand for plant-based meat (e.g., [10,11,12,13,14]) or milk (e.g., [15,16,17]). More recently, studies have expanded to include a wider range of categories beyond these dominant types, such as yogurt, seafood, and cheese (e.g., [18,19,20]). However, there is still little research specifically addressing plant-based eggs, particularly within the U.S. consumer context. A recent systematic review by Appiani et al. [21] indicates that research on plant-based eggs remains much more limited compared with plant-based meat and dairy alternatives. To date, virtually all of the research on plant-based eggs has been conducted outside of the U.S. Rondoni and colleagues examined consumer preferences for plant-based eggs as well as associations consumers make with plant-based eggs products in Italy and the United Kingdom [22,23]. They found that consumer preferences are influenced by both intrinsic (color, taste, ingredients, nutrient content) and extrinsic factors (price, packaging, product naming). Among Australian consumers, Jaeger, Chheang, and Ares [24] found that consumers had relatively low purchase intention towards plant-based eggs (and plant-based yogurt), though the specific ingredient composition could impact purchase intention. Additionally, the authors note that attitude towards plant-based diet and familiarity with plant-based products were important factors associated with purchase intention. More recently, Baxter et al. [25] examined how Canadian consumers perceived plant-based eggs, particularly focusing on sensory properties, emotional responses, and proposed usage. The study found that consumers’ sensory evaluation of plant-based eggs was lower compared with traditional eggs, which was a barrier to adoption.

As the popularity of plant-based eggs grows among producers and consumers, it becomes crucial to understand consumer perceptions of plant-based eggs as well as factors that may influence their purchasing decisions. While the literature on consumer preferences for plant-based eggs is growing, most existing studies primarily focus on general perception or acceptance without addressing what specific factors influence consumers’ purchase decisions. Furthermore, there are key gaps in the literature, particularly regarding the U.S. consumer context. Given that consumer perceptions and behavior towards plant-based products may be rooted from various factors like cultural norms, diet, and market contexts, findings from other countries may not be directly transferable to the U.S. context. For instance, Franzen and Vogl [26] show that the U.S. exhibits a lower level of environmental concern relative to some other developed countries, which could impact interest in plant-based products. Further, a prior study [27] found that discussions about plant-based products focus more on appearance in the U.S., while discussions are more centered around health and sustainability in European countries, like Switzerland. This highlights the need for U.S.-specific research given the growing interest and popularity of plant-based eggs. As such, the purpose of this study is to examine U.S. consumers’ perceptions of and purchase preferences for plant-based eggs. We study purchase preferences using experimental vignettes; specifically, we investigate how factors such as price, consumption location, and product form affect consumers’ likelihood of purchase for plant-based eggs. To our knowledge, this is one of the first studies to consider how factors such as where (home vs. restaurant) and how (main dish vs. ingredient to main dish) the product is consumed may impact purchasing decisions for plant-based eggs. Our results have important implications for the food industry in thinking about the broader context of the product purchase and positioning the product in a way that is most likely to draw in consumers.

## 2. Materials and Methods

### 2.1. Survey and Experimental Design

We designed an online survey to use with U.S. consumers to learn more about their perceptions of and preferences for plant-based eggs. The first portion of the survey included an experimental vignette exercise, designed to assess how different factors may affect consumers’ likelihood of purchase for plant-based eggs products. Vignettes refer to short descriptions of a person or a situation which contain precise references to highlight key factors that are believed to influence the decision-making or judgment-making processes of respondents [28]. The vignette methodology has been widely applied to social science fields, including social psychology, marketing, political science, and economics [29,30,31,32,33,34]. The vignette technique in survey research makes possible an analysis of the effects on people’s judgments by systematically varying the characteristics (attributes) used in the situation description [28]. The strength of the vignette method lies in its simplicity and straightforwardness. The survey questions were challenging for some participants to respond to, especially when they were tasked with unfamiliar food categories. Given the novelty of plant-based eggs in the market, many consumers may not have fully developed or realized their attitudes or perceptions towards these products. The vignette approach helps address this challenge by presenting respondents with detailed and relatable scenarios, which allows participants to engage more meaningfully with the questions. Additionally, our study focused on contextual attributes (which are described in detail in the next paragraph) rather than explicit product features such as brand name or front-of-package labels. Vignettes are particularly well suited for this purpose, allowing us to explore how consumers would react to different consumption settings rather than product-specific factors.

In this application, three attributes (price, consumption location, product form) were varied at two levels each, generating a total of eight vignette scenarios (2^3^ = 8). We included price as an attribute, as it has been recognized as one of the most critical factors influencing consumer preferences, perceptions, and adoption of plant-based protein alternatives [12,14,35,36]. Price levels were selected based on market prices for traditional and plant-based eggs available in the retail grocery market in the U.S., as well as menu prices for egg-based dishes in a restaurant setting. For consumption location, we considered both the in-home and away-from-home (restaurant) contexts. Past studies have examined how eating environment and location influence food choices and acceptance (e.g., [37,38]). When novel foods are introduced to consumers, there may be hesitancy to try cooking the novel foods at home, as they have limited experience using them; however, they may be willing to try them at a restaurant. Alternatively, if the consumer is uncertain about how much they will like the product, they may prefer to purchase the lower-cost option at the grocery store (relative to a restaurant) and try making the product at home. Lastly, we included the product form attribute, as the manner in which consumers will embrace various types of plant-based food remains uncertain and understudied in the current literature. Lusk et al. [39] found that consumers were more accepting of genetically engineered foods when they were processed (e.g., apple juice) compared with fresh (e.g., whole apple). More recently, Kim et al. [20] investigated the differences in consumer valuation of plant-based alternatives across fresh and processed plant-based seafood alternatives and found that consumers exhibited a higher willingness to pay for fresh plant-based seafood alternatives over processed alternatives. Plant-based eggs can be consumed either on their own (i.e., scrambled eggs) or as an ingredient in a more processed product (i.e., pancakes). We consider both product forms in our experimental design.

This study utilized a between-subject vignette design, in which respondents were randomly assigned to see one of the eight vignettes, and comparisons were made across the participants. The basic vignette is presented below. The text in brackets indicates attribute levels that varied across vignettes.

*Imagine you are planning to [eat breakfast at home; order breakfast at a restaurant]. You are considering [scrambled eggs; pancakes] that are made from plant-based eggs. The meal costs about [USD 4.99; USD 7.99]. How likely or unlikely would you be to purchase the product?*

We measured the likelihood of purchase using a 5-point Likert scale with response options of: I’d definitely purchase the plant-based eggs product; I’d probably purchase; I am uncertain; I’d probably not purchase; I’d definitely not purchase.

The second portion of the survey focused on assessing consumer perceptions of plant-based eggs. Consumer perceptions of plant-based eggs were divided into two categories, food attribute-related perceptions (e.g., taste, safety, price) and nutrition-related perceptions (e.g., calories, fat, cholesterol). Participants were asked to compare traditional eggs and plant-based eggs for each food and nutrition attribute using the following 5-point Likert scale: traditional eggs are much better; traditional eggs are better; traditional eggs and plant-based eggs are the same; plant-based eggs are better; plant-based eggs are much better. This scale is consistent with that used by Tonsor et al. [40] and Taylor et al. [41] when comparing plant-based and animal-based meats. We also asked consumers about their previous experience (if any) trying plant-based food products.

Lastly, the survey included questions regarding socio-demographic factors, such as gender, age, income, education level, race, geographical location (based on: (1) U.S. census region and (2) whether or not they reside in a metropolitan area), political views, household composition, participation in food assistance programs such as SNAP/WIC, and whether they have tried plant-based eggs previously.

### 2.2. Participant Recruitment and Data Collection

Data were collected between 15 May and 1 June 2023 through an online survey using the Qualtrics survey platform. Participants were recruited through the Qualtrics Research Panel service, and quota sampling was used so that the sample was representative of the U.S. population in terms of gender, age, and income. Individuals had to provide written consent to participate in the study in the first question of the online survey. Participation was limited to individuals aged 18 years and older who were fluent in English and physically located in the United States. The targeted sample size was 1600 respondents; thus, approximately 200 participants were randomized to each vignette scenario. The study was approved by the Institutional Review Boards at the University of Illinois Urbana-Champaign (IRB #23931) and Purdue University (IRB #2023-584). Table 1 summarizes the socio-demographic characteristics of respondents; we also report respondent characteristics for each vignette scenario in the Appendix A. The full dataset and coding files are available in the Supplementary Materials.

### 2.3. Data Analysis

To assess which factors influence the likelihood of purchase for plant-based eggs, we conducted an ordered logit regression analysis on a scale ranging from 1 (I’d definitely not purchase) to 5 (I’d definitely purchase). In the ordered logit regression, the intercept is represented by four threshold parameters (cut points). The estimated model is presented below.

*Purchase Likelihood* = *Intercept* + *β_p_* × *price* + *β_t_* × *product form* + *β_l_* × *consumption location* (1)

In this model, the price variable is continuous while the product form and consumption location variables are indicator variables. Product form takes a value of 1 if the product in the vignette scenario was pancakes and 0 if scrambled eggs. Similarly, the consumption location variable takes a value of 1 if the location in the vignette scenario was the restaurant setting and 0 for the home setting.

We extend Equation (1) by adding the interaction term between product form and consumption location. This addition allows us to explore for any joint effect between the two variables. Consumers may connect specific menu items with particular dining settings, influencing their decision to dine out or prepare meals at home. For example, when certain types of foods can be readily prepared at home, consumers might have a reduced inclination to seek those out in a restaurant setting.

We also extend the model by including socio-demographic variables to investigate individual heterogeneity in purchase likelihood. Socio-demographic characteristics have been extensively examined in consumer studies related to plant-based foods (see [35] for a review). However, there is no consensus on which characteristics are associated with consumers’ preferences for plant-based foods.

## 3. Results

### 3.1. Consumer Perception of Plant-Based Eggs

Recall that consumers were asked about their perceptions of plant-based eggs (relative to traditional eggs) on a variety of food-related and nutrition-related attributes. Consumer perceptions were assessed using a 5-point Likert scale. Average scores were computed for each food and nutrition attribute. An average score below 3 suggests that traditional eggs were perceived more favorably than plant-based eggs for a given attribute. Conversely, an average score above 3 implies that plant-based eggs were perceived to be better than traditional eggs. An average score of 3 indicates that both plant-based eggs and traditional eggs are perceived to be equivalent.

**Figure 1:**
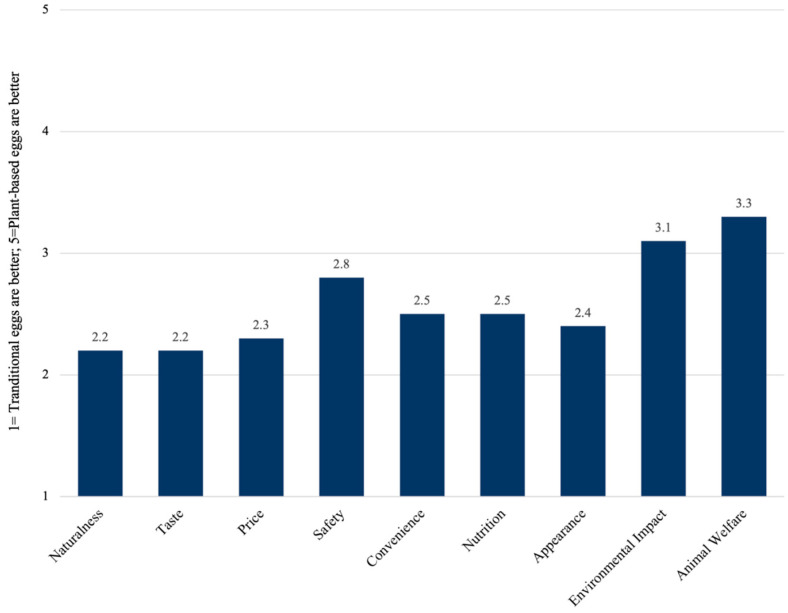
Food attribute perceptions of plant-based eggs relative to traditional eggs.

**Figure 2:**
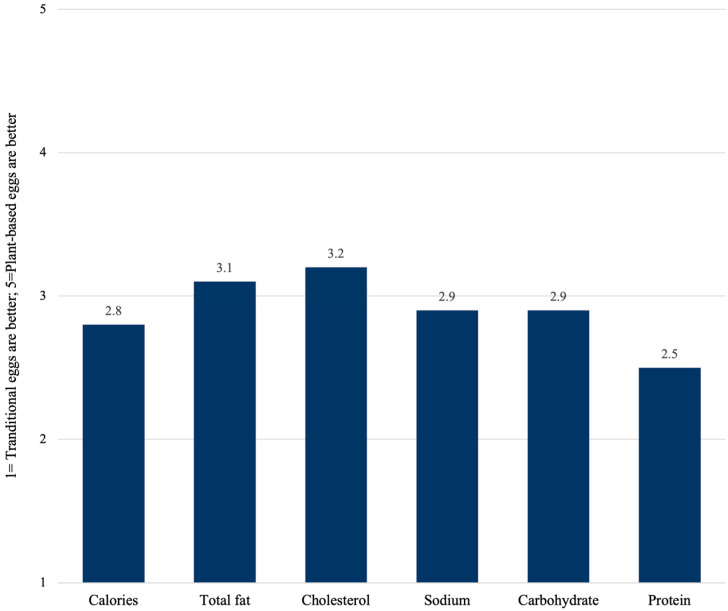
Nutrition attribute perceptions of plant-based eggs relative to traditional eggs.

Overall, participants rated traditional eggs higher than plant-based eggs for the majority of general food attributes (e.g., taste, price, convenience, nutrition, appearance); however, plant-based eggs were perceived to be better on the dimensions of environmental impact and animal welfare (see Figure 1). For the nutrition-related attributes, average scores were closer to three overall, indicating more parity between traditional and plant-based eggs (see Figure 2). Traditional eggs were perceived better on protein and had a slight edge in terms of calories, sodium, and carbohydrates, while plant-based eggs were perceived slightly better on the dimensions of total fat and cholesterol.

### 3.2. Experimental Vignette Results

Table 2 presents the summary statistics for the experimental vignette study. For each scenario, Table 2 displays the number of participants assigned, the scenario attributes (location, product form, price), and the average likelihood of purchase rating. Recall that the likelihood of purchase was assessed using a 5-point Likert scale (ranging from 1 = definitely not purchase to 5 = definitely purchase). The summary statistics indicate that a lower price point is associated with a higher likelihood of purchase. Additionally, purchase ratings tend to be higher for pancakes relative to scrambled eggs. Overall, the likelihood of purchase for most scenarios is above 3 (with the exception of scenario 2), suggesting participants are more positive than negative in terms of the likelihood of purchase, though the values near 3 indicate that many consumers are still uncertain about their purchase decision.

To examine which vignette factors were associated with likelihood of purchase, we estimated an ordered logistic regression, as specified in Equation (1). The results are presented in Table 3. The model 1 specification (vignette attributes only) indicates that price and product form were significant factors in consumers’ purchase likelihood ratings. Much like the summary statistics in Table 2 suggest, our results in Table 3 show that lower prices and the pancake product form (as opposed to scrambled eggs) were associated with a higher likelihood of purchase for the plant-based eggs product. We did not find a significant relationship between consumption location and likelihood of purchase in the base model specification.

In the model 2 specification, we introduced an interaction term between the product form and location variables to explore their joint effect. Like the model 1 results, we found that pancakes were preferred to scrambled eggs and location had a non-significant main effect. However, the interaction term between location and product emerged as marginally significant and negative (*p* < 0.10). Thus, while consumers had a greater likelihood of purchase for pancakes overall, there was a reduction in the likelihood of purchase when pancakes were being ordered in a restaurant setting (relative to home). Based on the magnitude of the interaction coefficient, this reduction only partially offsets the positive main effect of the pancakes product form—meaning pancakes made from plant-based eggs are more preferred than scrambled eggs made from plant-based eggs at home and in a restaurant, but the gap between the two is smaller in the restaurant setting.

In the model 3 specification, we built on model 2 by incorporating socio-demographic variables to explore individual heterogeneity. In this specification, we no longer found a significant interaction between product form and location, but there were some significant associations between socio-demographic variables and likelihood of purchase. Specifically, younger respondents (18–34 and 35–54 years) demonstrated a higher likelihood of purchase for plant-based eggs compared with those aged 55 years and older. Moreover, Black and African American respondents were more likely to purchase plant-based eggs products compared with respondents from other races. Geographic context also played a role, as respondents residing in metropolitan areas and the Northeast region of the U.S. showed a higher likelihood of purchase for plant-based eggs than their counterparts in non-metropolitan areas and the Southern region, respectively. Additionally, respondents with more liberal political views were more likely to purchase the product than those with conservative views. Household size also influenced purchase likelihood, with larger households being less likely to buy plant-based eggs than smaller households. Furthermore, respondents with children under 18 years old in their household displayed a higher likelihood of purchase compared with those without any children. Food assistance recipients also demonstrated a higher likelihood of purchase for plant-based eggs than non-recipients. Finally, previous experience with plant-based eggs significantly impacted purchase likelihood, as respondents who had tried the product before were more likely to purchase the plant-based eggs compared with those who had not. When looking at the magnitude of coefficients, previous exposure to plant-based eggs appears to be a driving factor in likelihood of purchase ratings.

## 4. Discussion

This study examined consumers’ perceptions of and likelihood of purchase for plant-based eggs using experimental vignettes. In terms of perceptions, we found that consumers generally perceived traditional eggs to be better than plant-based eggs on many food attributes, though plant-based eggs were viewed more favorably as it relates to environmental impact and animal welfare. Rondoni, Millan, and Asioli [23] similarly found that consumers associated plant-based eggs with environmental friendliness and animal welfare, and Nyambayo and Borusiak [42] concluded that environmental concern and moral issues connected with animal welfare were key motivations for consuming plant-based eggs. This also aligns with earlier studies [14,40,43], which discovered that consumers perceived plant-based meats as more environmentally friendly and better for animal welfare. The more favorable perceptions of traditional eggs on many attributes may come from familiarity or habitual consumption. Most consumers have extensive experience with traditional eggs, whereas plant-based eggs remain relatively new. This lack of familiarity may lead to uncertain expectations, especially when consumers are asked to make judgments in the absence of actual experience. Conversely, the more favorable perceptions of plant-based eggs (on environmental impact and animal welfare) might be driven by exposure to other existing plant-based products (e.g., plant-based meat, dairy) that emphasize those specific attributes in their packaging and marketing communications.

In terms of nutrition-related attributes, average scores hovered near the midpoint of the scale, indicating consumers view plant-based and traditional eggs as nearly equivalent on most nutrition attributes. Plant-based eggs were perceived as being slightly better than traditional eggs in terms of total fat and cholesterol. A review of plant-based products currently available on grocery store shelves confirms that claims such as “no cholesterol” or “cholesterol-free” are widely used to differentiate these products. A comparison of traditional and plant-based (vegan) eggs by Integris Health [44] confirms that plant-based eggs options are better than traditional eggs in terms of cholesterol and often better on fat content (especially saturated fat), but they can also be worse than traditional eggs on nutrients like sodium and may contain fewer vitamins and minerals.

Our results also revealed important insights into the factors associated with consumers’ likelihood of purchase for plant-based eggs. Price and product form were found to be significantly related to purchase likelihood; consumers were consistently more likely to purchase plant-based eggs when they were cheaper and when they were a product ingredient for pancakes rather than a final product (scrambled eggs). This is consistent with findings from Lusk et al. [39] that showed consumers were more accepting of a processed apple juice derived from genetic engineering relative to a whole apple. We recognize that consumers may have a general preference for pancakes over scrambled eggs, independent of the egg’s source (plant-based or conventional). This general preference for pancakes could influence the higher likelihood of purchase for plant-based eggs in that form, rather than being solely driven by the plant-based nature of the product. Additionally, because pancakes are typically seen as a dish where eggs are just one of several ingredients, while scrambled eggs are served on their own, consumers may perceive the quality and presence of the eggs differently. Multiple reviews [21,35,45,46] suggest that sensory properties of plant-based products, including eggs, are or are likely to be a barrier to consumption, which could partially explain the lower likelihood of purchase for scrambled eggs, as there are fewer ingredients to mask potential sensory issues relative to pancakes. Further research is needed to distinguish the specific impact of plant-based eggs from general preferences for pancakes and scrambled eggs by considering consumers’ base preferences for each dish.

Consumption location did not have a significant relationship with likelihood of purchase. This finding diverges from previous research somewhat; studies by Edwards et al. [37] and Garcia-Segovia et al. [38] found that consumers view foods as more acceptable in restaurant settings. While we did not find this result to be significant, the restaurant main effect coefficients trended in a positive direction. We also found that there exists a marginally significant joint effect (in model 2 specification in Table 3) between product form and location, suggesting that consumers’ purchase likelihood may vary depending on the location where the product is consumed. A positive main effect for the restaurant setting (though not significant) indicates that likelihood of purchase will be higher for both scrambled eggs and pancakes made from plant-based eggs, all else constant. However, we found a negative interaction effect between pancakes and the restaurant setting, which means pancakes made from plant-based eggs have a slight reduction in likelihood of purchase at restaurants. Plant-based scrambled eggs will not experience this same reduction in restaurants, but have a lower likelihood of purchase rating overall. One potential explanation for this finding is that consumers may believe pancakes are a food that can be easily prepared at home and are less worried about the impact of a less familiar plant-based egg as an ingredient relative to being a main dish. As a main dish, they may prefer to visit a restaurant in hopes of having a good product experience. That being said, the significance of the interaction between location and product form was weakened when socio-demographic variables were added to the model.

When looking at individual level differences in likelihood of purchase, we found that younger respondents showed a higher likelihood of purchase for plant-based eggs compared with their older (55 years or more) counterparts. This is consistent with previous findings related to plant-based meat. Multiple literature reviews [35,47] have highlighted that younger consumers have consistently been more accepting of alternative proteins, including plant-based meats. Additionally, we found that individuals who live in metropolitan areas and who are more liberal in their political views were more likely to purchase plant-based eggs. These findings also track with previous research related to alternative proteins. De Boer and Aiking [48] found that urban consumers were more accepting of alternative proteins, and multiple studies have suggested that individuals who identify as more liberal exhibit lower levels of food neophobia, making them more willing to purchase cultured meat [49,50]. While we find that many socio-demographic characteristics were associated with likelihood of purchase, our results also revealed that previous experience with plant-based eggs was a key driver of purchase likelihood. Other studies have also identified familiarity/experience with plant-based foods as a key factor in consumer acceptance (see reviews by [21,46,47]) and argue that constructs like familiarity, attitudes, and beliefs typically have stronger associations with purchase preferences compared with socio-demographic factors.

Based on our findings, there are important implications for various stakeholders in the food industry. For producers and retailers of plant-based eggs, understanding the factors that influence consumers’ purchase decisions can help optimize marketing strategies. Price sensitivity and product form should be considered when targeting specific consumer segments. For example, introducing plant-based eggs as an ingredient in a favorite product may be a more effective way to encourage new consumers to try the product. Additionally, the significance of environmental impact and animal welfare in consumers’ perceptions suggests that highlighting the sustainability aspects of plant-based eggs could be a key marketing strategy. This aligns with recommendations from Park and Namkung [51], who studied values, attitudes, and behaviors around plant-based foods in Korea. Boukid and Gagaoua [52] further note that label claims such as “animal-free” and “cholesterol-free” can also increase interest in plant-based eggs. Additionally, targeted marketing aimed at younger and urban consumers could help facilitate early-stage adoption and build broader market acceptance. Matharu et al. [53] suggest additional segmentation on dietary types (e.g., vegetarians, flexitarians) as well as knowledge and cooking skills around plant-based foods could also be beneficial. Policymakers can also benefit from this study’s findings, particularly concerning food environments. While the consumption location did not show significant impacts on purchase likelihood, the interaction between product form and location implies that interventions in food environments, such as restaurants and cafeterias, could influence consumer decision to choose plant-based options. Encouraging the availability of plant-based eggs options in such settings may facilitate healthier and more sustainable dietary choices.

Our study contributes to the emerging literature on plant-based eggs alternatives by specifically examining how contextual factors influence U.S. consumers’ purchase likelihood and by providing empirical evidence on how U.S. consumers evaluate plant-based eggs compared with traditional eggs. While our study offers new insights into the literature on plant-based eggs, there are many avenues for future research. One limitation of this study is that it did not ask consumers about their sensory perceptions for the products used in the vignettes. Several reviews [21,35,45,46,54] suggest sensory properties are a common barrier to adoption and consumption of plant-based foods. Therefore, future research should incorporate direct sensory evaluations and consider how sensory evaluations impact purchase behavior. Examining consumer response to intrinsic attributes, such as taste, texture, and smell, could provide a more comprehensive understanding of how consumers evaluate plant-based eggs and whether (and in what form) consumers will choose to consume them. Second, while this study identified attributes in which plant-based eggs were rated more favorably than traditional eggs, we did not directly test how much marketing those attributes could impact purchase intentions. More research is needed to explore the role of marketing interventions in promoting plant-based alternatives. Finally, this study was only conducted with U.S. consumers. While this is a contribution as the first U.S. study on consumer preferences for plant-based eggs, there may be concerns about whether results are generalizable to other countries. We find that perceptions around food and nutrition attributes of plant-based eggs were consistent with those in non-U.S. contexts; however, it is unclear whether consumers outside of the U.S. would prefer the pancake product form relative to scrambled eggs. In general, U.S. consumers have a higher consumption of processed foods relative to many other developed countries (see review [55]). We leave these issues to future research.

## 5. Conclusions

This study contributes to filling an existing gap in understanding consumer behavior in adopting plant-based foods and, particularly, plant-based eggs—which are understudied relative to plant-based meat and dairy alternatives. While consumers’ perceptions generally favored traditional eggs over plant-based eggs on many food and nutrition attributes, there were areas in which plant-based eggs had an advantage (e.g., environmental impact, animal welfare, and cholesterol content). These are likely attributes to emphasize in marketing efforts, Additionally, the vignette experiment underscores the importance of price and product form in influencing consumers’ purchase likelihood for plant-based eggs. Lower prices and using plant-based eggs as an ingredient (in this study, for pancakes) instead of a main dish were found to significantly increase purchase likelihood. Overall, the results of this study provide insights for policymakers, stakeholders in the food industry, and marketers. Understanding the drivers behind consumer behavior towards plant-based eggs can inform strategies for product development, pricing, and marketing, ultimately facilitating the transition towards more sustainable and ethical food choices.

## Figures and Tables

**Table 1:** Socio-demographic composition of U.S. consumer sample.

Variables	Definitions	Number (*n*)	Percentage/ Mean ± SD
Female (*n* = 1628)	1 if female; 0 otherwise	826	50.74%
Age (*n* = 1628)			
Age 18–34	1 if 18–34 years old; 0 otherwise	523	32.13%
Age 35–54	1 if 35–54 years old; 0 otherwise	529	32.49%
Age 55 and older	1 if 55 years or older; 0 otherwise	576	35.38%
Income (*n* = 1628)			
Income under USD 50 K	1 if annual income is less than USD 50,000; 0 otherwise	669	41.09%
Income USD 50–99 K	1 if annual income is USD 50,000–99,999; 0 otherwise	528	32.43%
Income USD 100 K and more	1 if annual income is USD 100,000 or more; 0 otherwise	431	26.47%
Education (*n* = 1627)	1 if obtained Bachelor’s degree or higher; 0 otherwise	674	41.40%
Race (*n* = 1627)			
White/Caucasian	1 if White/Caucasian; 0 otherwise	1200	73.71%
Black/African American	1 if Black/African American; 0 otherwise	216	13.27%
Other races	1 if races are not White/Caucasian nor Black/African American; 0 otherwise	211	12.96%
Geographic Region (*n* = 1628)			
Midwest	1 if Midwest; 0 otherwise	350	21.50%
Northeast	1 if Northeast; 0 otherwise	371	22.79%
West	1 if West; 0 otherwise	618	37.96%
South	1 if South; 0 otherwise	289	17.75%
Metropolitan (*n* = 1627)	1 if living in metropolitan area; 0 otherwise	754	46.34%
Political view (*n* = 1612)	1 if liberal; 5 if conservative		2.92 ± 1.31
Household size (*n* = 1628)	Number of household members		2.76 ± 1.44
Children in Household (*n* = 1628)	1 if children under 18 live in household; 0 otherwise	605	37.16%
SNAP (*n* = 1628)	1 if participating in a food assistance program such as SNAP or WIC; 0 otherwise	443	27.21%
Try PB Eggs (*n* = 1628)	1 if has tried plant-based eggs previously; 0 otherwise	550	33.85%

**Table 2:** Summary statistics for experimental vignettes (*N* = 1628).

Scenario	Number	Location	Product Form	Price	Mean Likelihood of Purchase
1	187	Home	Scrambled eggs	USD 4.99	3.16
2	207	Home	Scrambled eggs	USD 7.99	2.95
3	189	Home	Pancakes	USD 4.99	3.74
4	219	Home	Pancakes	USD 7.99	3.42
5	196	Restaurant	Scrambled eggs	USD 4.99	3.46
6	204	Restaurant	Scrambled eggs	USD 7.99	3.04
7	225	Restaurant	Pancakes	USD 4.99	3.60
8	201	Restaurant	Pancakes	USD 7.99	3.52

Note: Likelihood of purchase is assessed on a 5-point Likert scale (1 = definitely not purchase; 5 = definitely purchase).

**Table 3:** Ordered logistic regression results from experimental vignettes.

Variables	Model 1	Model 2	Model 3
Price	−0.103 *** (0.030)	−0.105 *** (0.030)	−0.131 *** (0.031)
Location: Restaurant ^a^	0.073 (0.088)	0.254 (0.127)	0.186 (0.131)
Product Form: Pancakes ^b^	0.502 *** (−0.089)	0.683 *** (0.127)	0.680 *** (0.131)
Location × Product Form		−0.354 * (0.177)	−0.277 (0.184)
Female ^c^			−0.123 (0.097)
Age 18–34 ^d^			0.971 *** (0.137)
Age 35–54 ^d^			0.599 *** (0.127)
Income under USD 50 K ^e^			−0.050 (0.135)
Income USD 50–99 K ^e^			−0.051 (0.127)
Education: Bachelor’s degree ^f^			0.002 (0.104)
White/Caucasian ^g^			0.237 (0.150)
Black/African American ^g^			0.716 *** (0.193)
Midwest ^h^			−0.104 (0.151)
Northeast ^h^			0.268 * (0.151)
West ^h^			0.014 (0.137)
Metropolitan ^i^			0.473 *** (0.099)
Political view			−0.238 *** (0.044)
Household size			−0.085 ** (0.041)
Children in Household ^j^			0.462 *** (0.132)
SNAP ^k^			0.357 *** (0.114)
Try PB Eggs ^l^			1.255 *** (0.111)
Number of Observations	1628	1628	1609 ^†^
Pseudo R^2^	0.009	0.010	0.1173

Notes: Standard errors are in parentheses. ***, **, and * denote statistical significance at the 1%, 5%, and 10% levels, respectively. Reference categories for indicator variables are denoted by superscripts as follows: ^a^ Home; ^b^ Scrambled eggs; ^c^ Non-female; ^d^ Age of 55 and older; ^e^ Income USD 100 K and above; ^f^ Other races; ^g^ Having less than a bachelor’s degree; ^h^ South; ^i^ Non-metropolitan; ^j^ No children under 18 in the household; ^k^ Participants who do not receive food assistance like SNAP/WIC; ^l^ Have not previously tried plant-based eggs. Political view is a continuous variable where 1 = liberal and 5 = conservative. Price and household size are also continuous variables. ^†^ For Model 3, 19 observations were dropped due to missing values associated with socio-demographic characteristics.

## Data Availability

The original contributions presented in the study are included in the article/Supplementary Materials; further inquiries can be directed to the corresponding author.

## Supplementary Materials

The following supporting information can be downloaded at: https://www.mdpi.com/article/10.3390/foods14101742/s1, File S1: Data; File S2: Variable Coding; File S3: Stata Code.

## Appendix A

**Table A1:** Sample characteristics by vignette scenario.

Variables	Scenario 1	Scenario 2	Scenario 3	Scenario 4	Scenario 5	Scenario 6	Scenario 7	Scenario 8	*p*-Value
Female	56.68	46.86	49.21	52.97	47.96	50.98	51.56	49.75	0.629
Age 18–34	33.16	29.47	33.86	34.70	30.10	32.35	32.00	31.34	0.953
Age 35–54	27.27	32.85	33.33	28.31	36.73	37.25	32.00	32.34	0.343
Age 54 and older	39.57	37.68	32.80	36.99	33.16	30.39	36.00	36.32	0.603
Income under USD 50 K	44.39	39.13	38.10	45.21	39.29	44.61	40.00	37.81	0.574
Income USD 50–99 K	36.36	28.50	34.92	30.14	36.73	27.45	32.44	33.83	0.334
Income USD 100 K and more	19.25	32.37	26.98	24.66	23.98	27.94	27.56	28.36	0.164
Bachelor’s degree	34.76	43.48	42.86	41.10	43.88	39.71	43.56	41.29	0.646
White/Caucasian	75.94	71.50	69.31	79.45	71.43	76.96	70.67	74.13	0.229
Black/African American	11.76	14.01	15.34	10.96	13.27	10.78	16.44	13.43	0.628
Other races	12.30	14.49	15.34	9.13	15.31	12.25	12.89	12.44	0.612
Midwest	16.58	19.81	25.40	17.35	17.86	24.02	25.33	25.37	0.072
Northeast	25.67	26.57	21.69	25.57	23.98	13.24	23.11	22.39	0.044
West	42.25	37.68	35.45	39.73	36.73	42.16	35.11	34.83	0.596
South	15.51	15.94	17.46	17.35	21.43	20.59	16.44	17.41	0.737
Living in metropolitan area	38.50	43.96	50.26	44.95	50.00	46.08	47.11	49.75	0.292
Children in Household	31.55	37.20	39.68	39.27	32.65	39.22	37.78	39.30	0.545
Food assistance participation	26.74	25.12	25.93	27.85	22.96	29.9	29.33	29.35	0.757
Have tried plant-based eggs	26.34	33.33	35.45	33.94	39.29	32.35	36.00	33.50	0.316
Political view	3.02	2.89	2.90	2.92	2.88	2.99	2.94	2.88	0.220
Household size	2.67	2.76	2.76	2.91	2.61	2.81	2.74	2.79	0.217

Note: *p*-values are derived from Chi-squared (for discrete variables) and ANOVA (for continuous variables) tests to assess if there are significant differences across vignette scenarios.

## References

[1] Crippa M., Solazzo E., Guizzardi D., Monforti-Ferrario F., Tubiello F.N., Leip A. (2021). Food Systems Are Responsible for a Third of Global Anthropogenic GHG Emissions. Nat. Food.

[2] Frezal C., Nenert C., Gay H. (2022). Meat Protein Alternatives: Opportunities and Challenges for Food Systems’ Transformation.

[3] Abín R., Laca A., Laca A., Díaz M. (2018). Environmental Assesment of Intensive Egg Production: A Spanish Case Study. J. Clean. Prod..

[4] FAO (2022). Thinking about the Future of Food Safety.

[5] The Plant Based Foods Association (2024). 2022 Plant-Based State of the Marketplace Summary Report. https://plantbasedfoods.org/latest/plant-based-foods-state-of-the-marketplace-2022-report.

[6] The Good Food Institute (2023). 2023 State of the Industry Report. https://gfi.org/state-of-alternative-proteins/.

[7] Global Market Insights Plant-Based Eggs Market Size—By Product, by Source, by Form, by Application, by Distribution Channel Analysis, Share, Growth Forecast, 2025–2034. https://www.gminsights.com/industry-analysis/plant-based-eggs-market.

[8] The Good Food Institute U.S. Retail Market Insights for the Plant-Based Industry. https://gfi.org/marketresearch/#eggs.

[9] Plant Based News Vegan Egg Demand Soars Amid US Egg Shortage. https://plantbasednews.org/news/economics/vegan-egg-demand-egg-shortage/.

[10] Katare B., Yim H., Byrne A., Wang H.H., Wetzstein M. (2023). Consumer Willingness to Pay for Environmentally Sustainable Meat and a Plant-Based Meat Substitute. Appl. Econ. Perspect. Policy.

[11] Michel F., Hartmann C., Siegrist M. (2021). Consumers’ Associations, Perceptions and Acceptance of Meat and Plant-Based Meat Alternatives. Food Qual. Prefer..

[12] Slade P. (2018). If You Build It, Will They Eat It? Consumer Preferences for Plant-Based and Cultured Meat Burgers. Appetite.

[13] Sogari G., Caputo V., Joshua Petterson A., Mora C., Boukid F. (2023). A Sensory Study on Consumer Valuation for Plant-Based Meat Alternatives: What Is Liked and Disliked the Most?. Food Res. Int..

[14] Van Loo E.J., Caputo V., Lusk J.L. (2020). Consumer Preferences for Farm-Raised Meat, Lab-Grown Meat, and Plant-Based Meat Alternatives: Does Information or Brand Matter?. Food Policy.

[15] Moss R., Barker S., Falkeisen A., Gorman M., Knowles S., McSweeney M.B. (2022). An Investigation into Consumer Perception and Attitudes towards Plant-Based Alternatives to Milk. Food Res. Int..

[16] Rombach M., Lucock X., Dean D.L. (2023). No Cow? Understanding US Consumer Preferences for Plant-Based over Regular Milk-Based Products. Sustainability.

[17] Slade P., Markevych M. (2024). Killing the Sacred Dairy Cow? Consumer Preferences for Plant-Based Milk Alternatives. Agribusiness.

[18] Jaeger S.R., Chheang S.L., Ares G. (2025). How Do Omnivore Consumers Perceive Plant-Based Alternatives to Yoghurt, Cheese, Eggs and Salmon? Comparison with Animal-Based Counterparts and Consideration of the Effect of Nutrition and Ingredient Information. Food Qual. Prefer..

[19] Etter B., Michel F., Siegrist M. (2024). Consumers’ Categorizations of Dairy Products and Plant-Based Milk, Yogurt, and Cheese Alternatives. Appetite.

[20] Kim D., Caputo V., Kilders V. (2023). Consumer Preferences and Demand for Conventional Seafood and Seafood Alternatives: Do Ingredient Information and Processing Stage Matter?. Food Qual. Prefer..

[21] Appiani M., Cattaneo C., Laureati M. (2023). Sensory Properties and Consumer Acceptance of Plant-Based Meat, Dairy, Fish and Eggs Analogs: A Systematic Review. Front. Sustain. Food Syst..

[22] Rondoni A., Grebitus C., Millan E., Asioli D. (2021). Exploring Consumers’ Perceptions of Plant-Based Eggs Using Concept Mapping and Semantic Network Analysis. Food Qual. Prefer..

[23] Rondoni A., Millan E., Asioli D. (2021). Consumers’ Preferences for Intrinsic and Extrinsic Product Attributes of Plant-Based Eggs: An Exploratory Study in the United Kingdom and Italy. Br. Food J..

[24] Jaeger S.R., Chheang S.L., Ares G. (2023). Beyond Plant-Based Alternatives to Milk and Meat: Product and Individual Variables Influence Purchase Intention for Plant-Based Yoghurt and Eggs. Food Qual. Prefer..

[25] Baxter L., Dolan E., Frampton K., Richelle E., Stright A., Ritchie C., Moss R., McSweeney M.B. (2024). Investigation into the Sensory Properties of Plant-Based Eggs, as Well as Acceptance, Emotional Response, and Use. Foods.

[26] Franzen A., Vogl D. (2013). Two Decades of Measuring Environmental Attitudes: A Comparative Analysis of 33 Countries. Glob. Environ. Change.

[27] Runte M., Guth J.N., Ammann J. (2024). Consumers’ Perception of Plant-Based Alternatives and Changes over Time. A Linguistic Analysis across Three Countries and Ten Years. Food Qual. Prefer..

[28] Alexander C.S., Jay Becker H. (1978). The Use of Vignettes in Survey Research. Public Opin. Q..

[29] Ambuehl S., Ockenfels A. (2017). The Ethics of Incentivizing the Uninformed: A Vignette Study. Am. Econ. Rev..

[30] Ellison B., Lusk J.L. (2018). Examining Household Food Waste Decisions: A Vignette Approach. Appl. Econ. Perspect. Policy.

[31] Epstein D., Mason A., Manca A. (2008). The Hospital Cost of Care for Stroke in Nine European Countries. Health Econ..

[32] Kapteyn A., Smith J.P., Van Soest A. (2007). Vignettes and Self-Reports of Work Disability in the United States and the Netherlands. Am. Econ. Rev..

[33] Kristensen N., Johansson E. (2008). New Evidence on Cross-Country Differences in Job Satisfaction Using Anchoring Vignettes. Labour Econ..

[34] Wilson N.L.W., Miao R., Weis C.S. (2019). When in Doubt, Throw It Out! The Complicated Decision to Consume (or Waste) Food by Date Labels. Choices.

[35] Onwezen M.C., Bouwman E.P., Reinders M.J., Dagevos H. (2021). A Systematic Review on Consumer Acceptance of Alternative Proteins: Pulses, Algae, Insects, Plant-Based Meat Alternatives, and Cultured Meat. Appetite.

[36] Tonsor G.T., Lusk J.L., Schroeder T.C. (2022). Market Potential of New Plant-Based Protein Alternatives: Insights from Four US Consumer Experiments. Appl. Econ. Perspect. Policy.

[37] Edwards J.S.A., Meiselman H.L., Edwards A., Lesher L. (2003). The Influence of Eating Location on the Acceptability of Identically Prepared Foods. Food Qual. Prefer..

[38] García-Segovia P., Harrington R.J., Seo H.S. (2015). Influences of Table Setting and Eating Location on Food Acceptance and Intake. Food Qual. Prefer..

[39] Lusk J.L., Mcfadden B.R., Rickard B.J. (2015). Which Biotech Foods Are Most Acceptable to the Public?. Biotechnol. J..

[40] Tonsor G.T., Lusk J.L., Schroeder T.C. (2021). Impacts of New Plant-Based Protein Alternatives on U.S. Beef Demand. https://www.agmanager.info/sites/default/files/pdf/PlantBasedProteinAlternatives_FullReport.pdf.

[41] Taylor H., Tonsor G.T., Lusk J.L., Schroeder T.C. (2022). Benchmarking US Consumption and Perceptions of Beef and Plant-Based Proteins. Appl. Econ. Perspect. Policy.

[42] Nyambayo I., Borusiak B., Bogueva D. (2024). Consumer Perceptions and Food.

[43] Bryant C., Sanctorum H. (2021). Alternative Proteins, Evolving Attitudes: Comparing Consumer Attitudes to Plant-Based and Cultured Meat in Belgium in Two Consecutive Years. Appetite.

[44] Integris Health Are Vegan Eggs Healthier Than Regular Eggs?. https://integrishealth.org/resources/on-your-health/2023/july/vegan-eggs.

[45] Rondoni A., Asioli D., Millan E. (2020). Consumer Behaviour, Perceptions, and Preferences towards Eggs: A Review of the Literature and Discussion of Industry Implications. Trends Food Sci. Technol..

[46] Szenderák J., Fróna D., Rákos M. (2022). Consumer Acceptance of Plant-Based Meat Substitutes: A Narrative Review. Foods.

[47] Giacalone D., Clausen M.P., Jaeger S.R. (2022). Understanding Barriers to Consumption of Plant-Based Foods and Beverages: Insights from Sensory and Consumer Science. Curr. Opin. Food Sci..

[48] De Boer J., Aiking H. (2011). On the Merits of Plant-Based Proteins for Global Food Security: Marrying Macro and Micro Perspectives. Ecol. Econ..

[49] Wilks M., Phillips C.J.C., Fielding K., Hornsey M.J. (2019). Testing Potential Psychological Predictors of Attitudes towards Cultured Meat. Appetite.

[50] Bryant C., Dillard C. (2019). The Impact of Framing on Acceptance of Cultured Meat. Front. Nutr..

[51] Park C.I., Namkung Y. (2024). Consumer Values, Attitudes, and Behavior towards Plant-Based Alternatives. Foods.

[52] Boukid F., Gagaoua M. (2022). Vegan Egg: A Future-Proof Food Ingredient?. Foods.

[53] Matharu G.K., von der Heidt T., Sorwar G. (2024). Consumer Behavior toward Plant-Based Foods: A Theoretical Review, Synthesis and Conceptual Framework. Br. Food J..

[54] Khalifa I., Li Z., Nawaz A., Walayat N., Sobhy R., Jia Y., Korin A., Zou X., Maqsood S. (2025). Recent Innovations for Improving the Techno-Functional Properties of Plant-Based Egg Analogs and Egg-Mimicking Products to Promote Their Industrialization and Commercialization. Compr. Rev. Food Sci. Food Saf..

[55] Marino M., Puppo F., Del Bo’ C., Vinelli V., Riso P., Porrini M., Martini D. (2021). A Systematic Review of Worldwide Consumption of Ultra-Processed Foods: Findings and Criticisms. Nutrients.

